# Coloenterostomy performed endoscopically using through-the-scope twin clips in a porcine model

**DOI:** 10.1055/a-2085-0199

**Published:** 2023-06-22

**Authors:** Qiang Zhang, Zhen Wang, Chudi Chen, Side Liu

**Affiliations:** Guangdong Provincial Key Laboratory of Gastroenterology, Department of Gastroenterology, Nanfang Hospital, Southern Medical University, Guangzhou, Guangdong Province, China

A novel through-the-scope twin clip or dual action tissue closure device (TTS-TC or DAT Closure Device; Micro-Tech Co. Ltd., Nanjing, China) was first reported by us 1 2 . We have previously performed endoscopic gastric bypass using TTS-TCs in a live pig 3 . We have now for the first time performed coloenterostomy, similarly to colonic–small intestinal bypass, in a live pig. The operation steps are shown in Video 1 .

**Video 1** The colon, approximately 20 cm away from the anus, is anastomosed to the small intestine using through-the-scope twin clips to create an endoscopic coloenterostomy in a live pig.


First, in the colon approximately 20 cm away from the anus, a colonic opening was made into the abdominal cavity (opening 1) using endoscopic full-thickness resection ( Fig. 1 a ). Next, a TTS-TC was delivered into the abdominal cavity through the endoscope working channel, and the small intestinal wall was clamped using one side of the TTS-TC. The clamped intestine was pulled through opening 1 into the colon using the TTS-TC, then the colonic mucosa was clamped using the other side of the TTS-TC to anchor the intestine that had been pulled through into the colon ( Fig. 1 b ). A longitudinal opening (opening 2) was then made in the small intestine anchored in the colon ( Fig. 1 c ). The intestinal wall at the edge of opening 2 was clamped using one side of another TTS-TC and pulled close to the colon at opening 1, before openings 1 and 2 were clamped together using the other side of the TTS-TC ( Fig. 1 d ). Further TTS-TCs were used to continue joining openings 1 and 2, then traditional TTS clips were used to close the remaining wound. A total of nine TTS-TCs and 12 TTS clips were used. The total operation time was 75 minutes. Cephalosporins were administered intramuscularly for 1 week postoperatively.

**Fig. 1:**
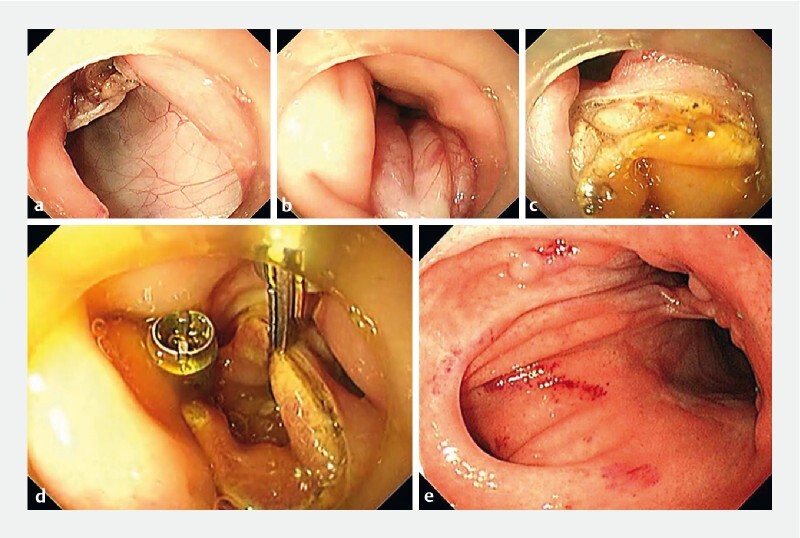
The steps in creation of an endoscopic coloenterostomy, anastomosing the colon approximately 20 cm away from the anus to the small intestine, were: **a** creation of opening 1 in the colonic wall by endoscopic full-thickness resection; **b** clamping of the small intestinal wall with one clip of a through-the-scope twin clip (TTS-TC), which was then pulled into the colonic lumen where it was anchored using the second clip of the TTS-TC; **c** creation of opening 2 in the small intestinal wall; **d** clamping together of the intestinal walls at the edges of openings 1 and 2 using further TTS-TCs; **e** successful creation of the coloenterostomy with good healing of the wound 2 months postoperatively.

At 2-month follow-up, no perforation or bleeding had occurred, the wound was healed and the TTS-TCs had fallen off spontaneously. The coloenterostomy had been successfully created ( Fig. 1 e ); however, further study is needed to optimize the procedure and evaluate its safety and feasibility.

Endoscopy_UCTN_Code_TTT_1AQ_2AF

## Notes

Endoscopy E-Videos

https://eref.thieme.de/e-videos
*E-Videos* is an open access online section of the journal *Endoscopy* , reporting on interesting cases and new techniques in gastroenterological endoscopy. All papers include a high-quality video and are published with a Creative Commons CC-BY license. Endoscopy E-Videos qualify for HINARI discounts and waivers and eligibility is automatically checked during the submission process. We grant 100% waivers to articles whose corresponding authors are based in Group A countries and 50% waivers to those who are based in Group B countries as classified by Research4Life (see: https://www.research4life.org/access/eligibility/ ). This section has its own submission website at https://mc.manuscriptcentral.com/e-videos
